# Correlation of OSCE performance and point-of-care ultrasound scan numbers among a cohort of emergency medicine residents

**DOI:** 10.1186/s13089-019-0118-7

**Published:** 2019-03-05

**Authors:** Youyou Duanmu, Patricia C. Henwood, Sukhjit S. Takhar, Wilma Chan, Joshua S. Rempell, Andrew S. Liteplo, Viktoria Koskenoja, Vicki E. Noble, Heidi H. Kimberly

**Affiliations:** 1 Department of Emergency Medicine, Stanford University School of Medicine, 900 Welch Road Suite 350, Palo Alto, CA 94304 USA; 2 Department of Emergency Medicine, Brigham and Women’s Hospital, Boston, MA USA; 3 Department of Emergency Medicine, Mills-Peninsula Medical Center, Burlingame, CA USA; 4 Department of Emergency Medicine, Hospital of the University of Pennsylvania, Philadelphia, PA USA; 5 Department of Emergency Medicine, Cooper University Hospital, Camden, NJ USA; 6 Department of Emergency Medicine, Massachusetts General Hospital and Harvard Medical School, Boston, MA USA; 7 Department of Emergency Medicine, UP Health System-Marquette, Marquette, MI USA; 8 Department of Emergency Medicine, University Hospitals-Cleveland Medical Center, Cleveland, OH USA

**Keywords:** Education, Point of care, Ultrasound, Competency

## Abstract

**Background:**

Point-of-care ultrasound (POCUS) is an important clinical tool for a growing number of medical specialties. The current American College of Emergency Physicians (ACEP) Ultrasound Guidelines recommend that trainees perform 150–300 ultrasound scans as part of POCUS training. We sought to assess the relationship between ultrasound scan numbers and performance on an ultrasound-focused observed structured clinical examination (OSCE).

**Methods:**

This was a cross-sectional cohort study in which the number of ultrasound scans residents had previously performed were obtained from a prospective database and compared with their total score on an ultrasound OSCE. Ultrasound fellowship trained emergency physicians administered a previously published OSCE that consisted of standardized questions testing image acquisition and interpretation, ultrasound machine mechanics, patient positioning, and troubleshooting. Residents were observed while performing core applications including aorta, biliary, cardiac, deep vein thrombosis, Focused Assessment with Sonography in Trauma (FAST), pelvic, and thoracic ultrasound imaging.

**Results:**

Twenty-nine postgraduate year (PGY)-3 and PGY-4 emergency medicine (EM) residents participated in the OSCE. The median OSCE score was 354 [interquartile range (IQR) 343–361] out of a total possible score of 370. Trainees had previously performed a median of 341 [IQR 289–409] total scans. Residents with more than 300 ultrasound scans had a median OSCE score of 355 [IQR 351–360], which was slightly higher than the median OSCE score of 342 [IQR 326–361] in the group with less than 300 total scans (*p* = 0.04). Overall, a LOWESS curve demonstrated a positive association between scan numbers and OSCE scores with graphical review of the data suggesting a plateau effect.

**Conclusion:**

The results of this small single residency program study suggest a pattern of improvement in OSCE performance as scan numbers increased, with the appearance of a plateau effect around 300 scans. Further investigation of this correlation in diverse practice environments and within individual ultrasound modalities will be necessary to create generalizable recommendations for scan requirements as part of overall POCUS proficiency assessment.

## Background

Point-of-care ultrasound (POCUS) has become an important clinical tool across a variety of medical specialties [1–5]. Proficiency in POCUS is especially vital to the practice of emergency medicine (EM) [6–11]. Since 2012, the Accreditation Council for Graduate Medical Education (ACGME) has designated the use of ultrasound for diagnosing emergent medical conditions, critical care and trauma resuscitation, and procedural guidance as 1 of 23 milestone competencies for EM residents [8]. The 2016 American College of Emergency Physicians (ACEP) policy statement on emergency ultrasound advises that a trainee should perform 25–50 ultrasounds in each of the core applications and a total of 150–300 scans as part of POCUS training [9].

It has been suggested that the completion of a predetermined number of ultrasounds correlates with proficiency in clinical practice; however, there remains significant variability in the number of scans required by different training programs [12, 13]. There are data showing that residents who performed greater than 150 ultrasounds scored significantly higher on a written ultrasound examination [14]. However, a more recent consensus statement recommended that 150 scans may not be sufficient as a competency benchmark, but should be regarded as a minimum standard beyond which other measures of competency should be assessed [15]. A 2017 survey of 539 EM residents found that residents believed an average of 325 scans is required for proficiency [16].

Medical training programs have adapted the observed structured clinical examination (OSCE) to evaluate competency in a variety of applications [17–21]. The OSCE is an especially useful tool for medical skills that involve a combination of technical and knowledge-based aptitude. Its use is recommended by the ACEP policy statement to assess for ultrasound competency, but to our knowledge there is no prior data comparing OSCE performance to overall scan numbers [9].

Ultrasound is a multi-modal skill set requiring complex methods of competency assessment. In this study, we sought to assess whether there was a relationship between the number of ultrasounds previously performed by senior EM residents and their performance on a standardized ultrasound OSCE.

## Methods

This was a cross-sectional cohort study in which the number of ultrasound scans residents had previously performed was obtained from a prospective database and compared to total scores on an ultrasound OSCE. A modified version of a previously published ultrasound OSCE was given to all 29 postgraduate year (PGY)-3 and PGY-4 residents at a single academic emergency medicine residency program that spans two institutions [17, 22, 23]. The OSCE took place in a simulation center using standardized patients as models. It consisted of standardized questions testing image acquisition and interpretation with points for technique, image quality, and correct interpretation of anatomy. Twelve ultrasound fellowship trained emergency physicians served as evaluators. Residents were observed performing aorta, biliary, cardiac, deep vein thrombosis, Focused Assessment with Sonography in Trauma (FAST), pelvic, and thoracic ultrasounds, which are included in the ACEP core emergency ultrasound applications. The total possible score for the OSCE was 370. Based on the total OSCE score and overall evaluator impression, residents were given an OSCE general competency score from 1 to 5 and those with a score of 2 or below were provided individualized remediation. To assess inter-rater reliability, 11 residents had two evaluators independently grade their OSCE performance. For those 11 subjects, the mean of their two OSCE scores was used in the general analysis.

The number of ultrasound scans the residents had performed was obtained from a prospective database and included ultrasounds performed clinically and during the 1-week PGY-1 and 2-week PGY-2 ultrasound rotations. Scans were evaluated in total and by application type. None of the residents had participated in an outside ultrasound rotation. The scans logged in the database had all been reviewed for quality assurance by ultrasound-trained faculty.

Data were analyzed using Stata 14.2 (Stata Corporation, College Station, TX). A locally weighted scatter plot smoothing (LOWESS) method was used to visually estimate the trend between OSCE score and previously performed ultrasound scan numbers. A Wilcoxon rank-sum test was used to assess for differences in OSCE score by PGY year. A two-sample *t* test was used to assess for differences in OSCE score at a scan number cutoff of 300, because this appeared to be the region of plateau of the LOWESS curve as well as the upper range of recommended scan numbers based on the ACEP guidelines. Intraclass correlation (ICC) with one-way random effects was used to assess inter-rater reliability for those OSCEs that had two evaluators. The study was deemed exempt from review by the Partners Healthcare Institutional Review Board.

## Results

**Fig. 1:**
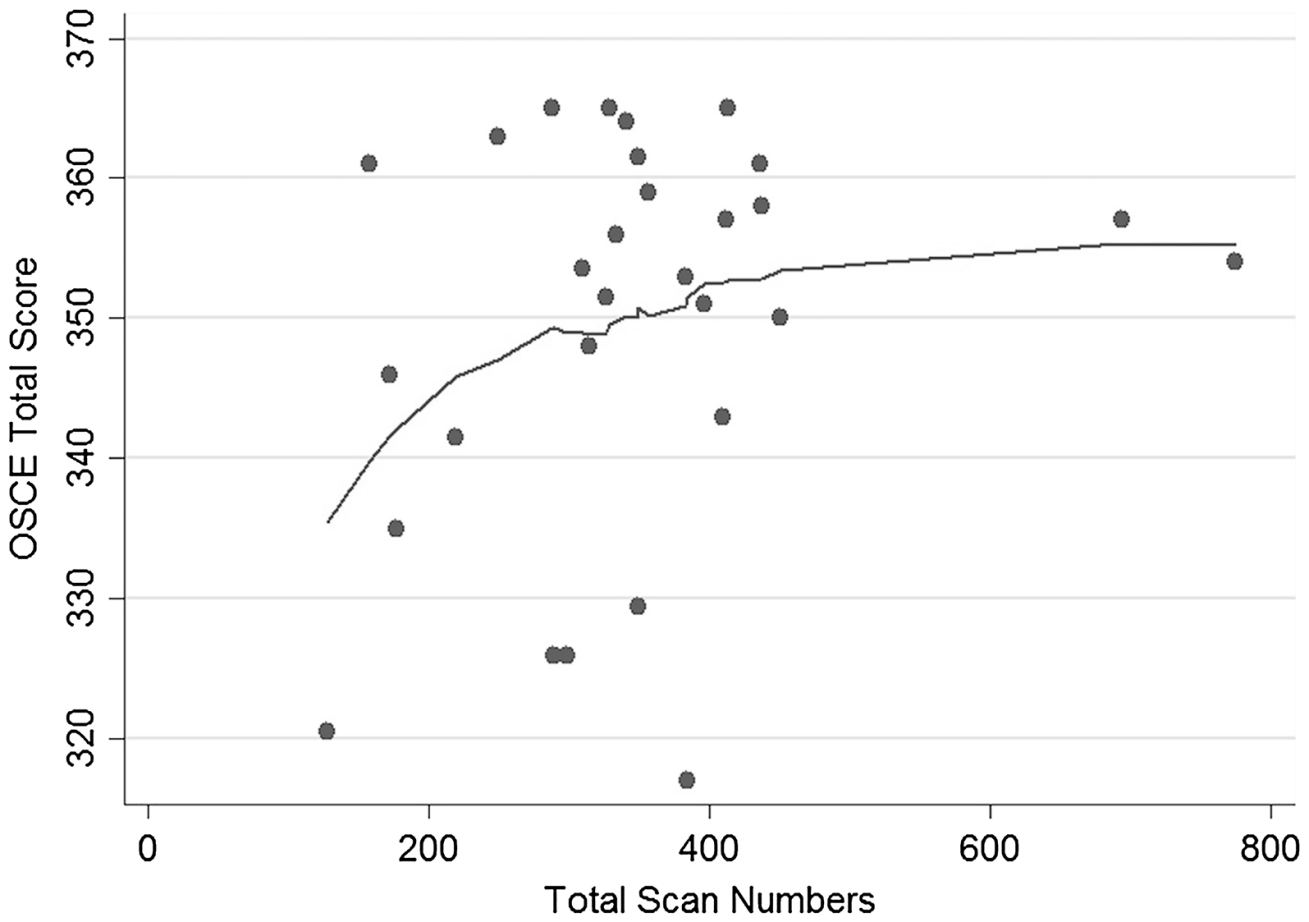
Scatter plot of observed structured clinical exam (OSCE) total score vs. total ultrasound scan numbers fit with locally weighted scatter plot smoothing (LOWESS) curve

All 29 senior residents in our program participated in the OSCE, including 15 PGY-3 and 14 PGY-4 residents. The median OSCE score for all participants was 354 [interquartile range (IQR) 343–361]. Residents had performed a median of 341[IQR 289–409] total scans, including 105 [IQR 87–120] cardiac, 79 [IQR 65–96] FAST, 48 [IQR 36–64] thoracic, 15 [IQR 12–10] pelvic, 16 [IQR 10–21] biliary, 15 [IQR 13–19] aorta and 3 [IQR 1–5] deep vein thrombosis. The LOWESS smoother curve suggested a pattern of increase and then a plateau in OSCE total score as total scan numbers increased (Fig. 1). Residents who had previously performed more than 300 scans had a slightly higher median OSCE score (355 [IQR 350–360]) than residents who had performed fewer than or equal to 300 scans (342 [IQR 326–361]), *p* = 0.04. The median OSCE score for PGY-3 residents was 354 [IQR 346–364], while the median score for PGY-4 residents was 352 [IQR 335–359], which was not significantly different (*p* = 0.43). There was a moderate ICC of 0.61 [CI 0.08–0.88], *p* = 0.01, for the 11 residents who had two evaluators.

## Discussion

Ultrasound is a multi-faceted skill that involves acquisition of images, interpretation of scans, and knowledge of how to incorporate findings into clinical practice. The evaluation of ultrasound competency also requires a multi-modal approach. OSCEs have been found to be a useful tool for measuring ultrasound proficiency, as both scanning technique and interpretation can be assessed in real time [17–20].

The results of this small single residency program study demonstrate that a higher number of scans performed were associated with higher performance on the OSCE with graphical review of the data suggesting a plateau around 300 scans. Defining a minimum number of scans required to attain competency can be challenging, and the current ACEP recommendation of 150–300 scans is a wide target. Our data intimates that within the suggested range of ultrasounds recommended by ACEP, there may be a plateau effect at the higher range of this requirement, but further investigation should be aimed at determining whether this plateau is consistent in different cohorts or rather may be institutionally dependent and vary with training methodologies. We found that in our cohort, a cutoff of 300 overall scans showed a small division in OSCE performance between residents, although it is unclear if this is clinically significant.

A recent study by Blehar et al. suggested that ultrasound image accuracy improved with the number of scans performed up to a “plateau” point where additional scan numbers did not greatly increase accuracy [24]. Our LOWESS curve of OSCE score and prior scan numbers also suggests an asymptotic pattern. This is similar to the concept outlined in the Pusic et al. paper on learning curves, in which medical training is correlated with rising skill level until competence is gained, after which increasing proficiency becomes more subtle despite additional practice [25]. Due to the varied complexity of ultrasound modalities, Blehar et al. found that proficiency for different ultrasound applications plateaued at different scan numbers [24]. We assessed scan proficiency as an aggregate, but were unable to analyze separate proficiency for each modality due to limited sample size.

It would also be valuable to identify a scan number requirement for credentialing physicians who are currently practicing. However, it is unclear if data obtained from emergency medicine residency training programs would be applicable to physicians who did not have structured ultrasound teaching during residency, or whether the plateau effect would differ based on the practice setting. Further investigation to examine the performance of competency assessment tools in a multi-center observational study across a variety of settings is indicated to better characterize the relationship between ultrasound numbers and observed performance in aggregate and within modalities.

This study has several limitations. It was performed at a single residency program with a small sample size and may not be generalizable outside of our institution or beyond emergency medicine. Although OSCE performance visually appeared to plateau around 300 scans, the clinical significance of this number is unclear. Only one resident had less than 150 scans and thus we did not evaluate a cutoff value of 150 scans. Residents had performed a larger percentage of cardiac and FAST examinations than the other modalities. The pattern of OSCE performance and plateau as an aggregate may be affected by this uneven scan number distribution, which included several modalities with median scan numbers below the ACEP-recommended benchmark for individual ultrasound applications [9]. Absolute number of ultrasounds performed may not be a reliable measure of a resident’s comprehensive ultrasound education, as it does not include instruction during the ultrasound elective, informal clinical teaching or exposure to a variety of pathology. Furthermore, residents’ ultrasounds were completed under the supervision of ultrasound-certified attending physicians and were often accompanied by real-time teaching and quality assurance review. There is a possibility that the database underrepresents actual scanning activity considering scans performed outside of the emergency department or those not properly documented would not have been captured.

The OSCE was created by the ultrasound faculty at our institution and has not been independently validated, although it had gone through an iterative process of improvement and has been used in multiple different clinical contexts over the past 4 years [17, 22, 23]. There was only moderate reliability between faculty who administered OSCE to the same resident, which indicates that the OSCE may require additional standardization to be a reliable measure of ultrasound proficiency. Small sample size precluded sub-group analysis of correlation between OSCE score and prior scan numbers for the individual ultrasound applications.

## Conclusion

The results of this small single residency program study suggest a pattern of improvement in OSCE performance as scan numbers increased, with the appearance of a plateau effect around 300 scans. While these are interesting findings for the assessment of ultrasound competency, additional investigation of this correlation in diverse practice environments and within individual ultrasound modalities will be necessary to create generalizable recommendations for scan requirements needed as part of overall POCUS competency assessment.

## Abbreviations

POCUS: point-of-care ultrasound
EM: emergency medicine
OSCE: observed structured clinical exam
FAST: Focused Assessment with Sonography in Trauma
PGY: postgraduate year
IQR: interquartile range
ACEP: American College of Emergency Physicians
ACGME: Accreditation Council for Graduate Medical Education
LOWESS: locally weighted scatter plot smoothing
ICC: intraclass correlation
CI: confidence interval
